# The complete chloroplast genome sequence of medicinal fern *Polypodiodes niponica* (Polypodiaceae)

**DOI:** 10.1080/23802359.2018.1491343

**Published:** 2018-07-11

**Authors:** Minghui Zhang, Haoyun Xiao, Shanshan Liu, Shufeng Li, Zhen Wang, Ting Wang, Yingjuan Su

**Affiliations:** a School of Life Sciences, Sun Yat-sen University, Guangzhou, China;; b College of Life Sciences, Nanjing Agricultural University, Nanjing, China;; c College of Life Sciences, South China Agricultural University, Guangzhou, China;; d Research Institute of Sun Yat-sen University in Shenzhen, Shenzhen, China

**Keywords:** *Polypodiodes niponica*, medicinal fern, chloroplast genome, phylogenetic analysis

## Abstract

The complete chloroplast genome of *Polypodiodes niponica* has been determined. It is a double stranded circular DNA of 152,551 bp length, containing two inverted repeat (IR, 24,643 bp each) regions, separated by large single-copy (LSC, 81,506 bp) and small single-copy (SSC, 21,759 bp) regions, respectively. The genome contains 131 genes, including 88 protein-coding genes, 35 tRNA genes, and eight rRNA genes. The total GC content of the chloroplast genome is 42.4%. ML phylogenetic analysis revealed that *P. niponica* was most closely related to *Lepisorus clathratus.*

*Polypodiodes niponica*, commonly known as *Polypodium niponicum* Mett. or *Marginaria niponica* Nakai, is a polypodiaceous fern. It is epiphytic on tree trunks or on rocks with elevation between 300–1800 m. Its rhizome is gray-green, long creeping, and sparsely covered with whitish bloom and scales (Zhang et al. 2013). The plant is extensively distributed in Japan and China such as Anhui, Fujian, Gansu, Guangdong etc. In China, it is not only an edible fern (Liu et al. 2012), but also an important medical plant known as “Shican” which is used in clearing heat and promoting diuresis (Wang et al. 2009). As a key genus in Polypodioideae, *Polypodiodes* plays an important role in exploring the phylogenetic relationships among *Polypodiastrum*, *Schellolepis*, and *Goniophlebium*, which occurs a great controversy (Schneider et al. 2004; Lu and Li 2006). Hence, the acquirement of whole chloroplast (cp) genome of *Polypodiodes niponica* will contribute to further development of the medicinal resources and survey phylogeny of the family Polypodioideae.

Total genomic DNA was isolated from fresh leaves sampled from the South China Botanical Garden (23°11′3.56″N, 113°21′43.28″E) using Tiangen Plant Genomic DNA Kit (Tiangen Biotech Co., Beijing, China). The specimen is stored in Herbarium of Sun Yat-sen University (SYS; voucher: *SS Liu 20161013*). On an average 300 bp DNA fragment was obtained using Covaris M220 (Covaris Inc., MS, USA), which was further used to construct genomic library. Sequencing was performed on Illumina Hiseq 2500. Trimmomatic v0.32 was employed to filter and trim low-quality reads (Bolger et al. 2014). After visual evaluation by FastQC v0.10.0 (Andrews 2010), we assembled high-quality clean reads into the chloroplast genome using Velvet v1.2.07 (Zerbino and Birney 2008) and annotated with DOGMA (Wyman et al. 2004) and tRNAscan-SE (Schattner et al. 2005). Finally, BLASTX and BLASTN were used to validate the location of encoding genes and RNAs. The complete chloroplast genome sequences of 11 ferns (*Equisetum arvense* as outgroup) were aligned by MAFFT (Katoh et al. 2002) and phylogenetic tree was inferred based on the maximum likelihood (ML) analysis using RAxML v.8.0 with 1000 bootstrap replicates (Stamatakis 2014).

The complete chloroplast genome of *P. niponica* is a double stranded circular DNA with 152, 551 bp length, containing two inverted repeat (IR) regions of 24,643 bp each, separated by large single-copy (LSC) and small single-copy (SSC) regions of 81,506 bp and 21,759 bp, respectively (GenBank accession number: MH319944). A total of 131 functional genes are successfully annotated, including 88 protein-coding genes (PCGs), 35 tRNA genes and eight rRNA genes. Among them, 14 are duplicated in the IR regions, including four PCGs, six tRNA genes and four rRNA genes. The GC content of the whole chloroplast genome is 42.4%, which is higher than that of LSC and SSC (41.5% and 39.0%, correspondingly). The IR region possesses the highest GC content (45.3%). Furthermore, there are 18 intron-containing genes, almost all of which are single-intron genes except for *ycf3*, *clpP*, and *rps12* with two introns. ML tree indicated that *P. niponica* was most closely related to *Lepisorus clathratus* with high support value (Figure 1). The chloroplast genome of *P. niponica* provides valuable molecular data for development of the medical fern and phylogeny of ferns.

**Figure 1.**
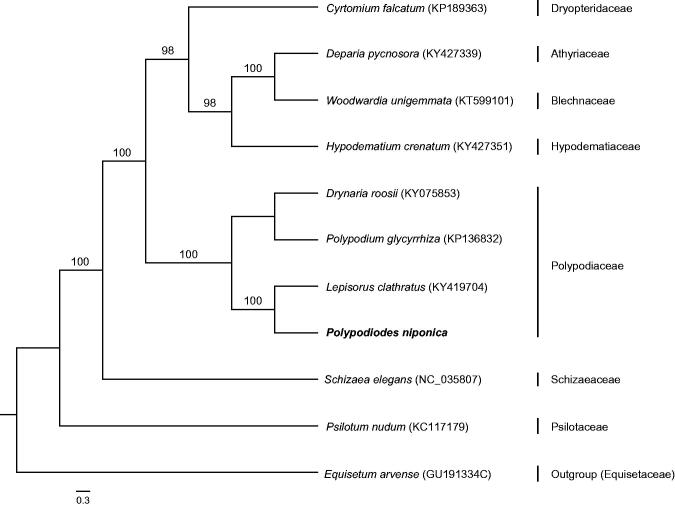
ML phylogenetic tree based on the complete chloroplast genome sequence of *P. niponica* and other ten ferns download from NCBI including *Equisetum arvense* as outgroup.
